# User Character Strengths and Engagement Prediction on a Digital Mental Health Platform for Young People: Longitudinal Observational Study

**DOI:** 10.2196/73793

**Published:** 2025-08-25

**Authors:** Alicia J Smith, Shaminka N Mangelsdorf, Simon T E Baker, Javad Jafari, Mario Alvarez-Jimenez, Caitlin Hitchcock, Shane Cross

**Affiliations:** 1 Melbourne School of Psychological Sciences, The University of Melbourne, University of Melbourne, Melbourne, Australia; 2 MRC Cognition and Brain Sciences Unit, University of Cambridge, 15 Chaucer Road, Cambridge, CB2 7EF, United Kingdom, 44 1223355294; 3 Orygen Youth Health, Parkville, Australia; 4 Centre for Youth Mental Health, University of Melbourne, Melbourne, Australia; 5 NovoPsych, Melbourne, Australia

**Keywords:** digital mental health interventions, engagement, persuasive systems design, personalization, self-determination theory, behavior change, measurement-based care, personality, mental health

## Abstract

**Background:**

Mental ill health is a leading cause of disability worldwide, but access to evidence-based support remains limited. Digital mental health interventions offer a timely and low-cost solution. However, improvements in clinical outcomes are reliant on user engagement, which can be low for digital interventions. User characteristics, including demographics and personality traits, could be used to personalize platforms to promote longer-term engagement and improved outcomes.

**Objective:**

This study aims to investigate how character strengths, a set of positive personality traits, influence engagement patterns with moderated online social therapy, a national digital mental health platform offering individualized, evidence-based digital mental health treatment for young people aged 12‐25 years.

**Methods:**

Data from 6967 young people who enrolled with moderated online social therapy between August 2021 and July 2023 were analyzed. Longitudinal analyses were used to investigate whether scores on 3-character strength dimensions (“social harmony,” “positive determination,” and “courage and creativity”) were associated with (1) an accelerated or decelerated rate of dropout from the platform and (2) patterns of engagement over the first 12 weeks following onboarding. Engagement metrics were time spent on the platform, number of sessions on the platform, use of the embedded social network, and messages with the clinical team.

**Results:**

On average, young people used the platform for 72.64 (SD 106.64) days. The 3-character strengths were associated with distinct engagement patterns during this time. Individuals scoring higher on “social harmony” demonstrated an accelerated dropout rate (coefficient=−0.15, 95% CI −0.26 to −0.04; *P*=.008). Interestingly, higher scores on this character strength were associated with high rates of initial engagement but a more precipitous decline in platform use over the first 12 weeks, in terms of time spent on the platform (β=−.01; SE 0.00; *t*_2748_=−5.05; *P*<.001) and the number of sessions completed (β=−.00; SE 0.00; *t*_2837_=−2.26; *P*=.02). In contrast, higher scores on “positive determination” and “courage and creativity” predicted more modest initial platform use but steadier engagement over time, in terms of time spent on the platform (“positive determination”: β=.01; SE 0.00; *t*_2748_=4.05; *P*<.001 and “courage and creativity”: β=.01; SE 0.00; *t*_2748_=2.66; *P*=.008). Contrary to our predictions, character strengths did not predict use of the embedded social network or the number of messages sent to the clinical team.

**Conclusions:**

Our findings illustrate how character strengths predict distinct engagement trajectories on a digital mental health platform. Specifically, individuals higher on “social harmony” showed high initial engagement that quickly declined, while those higher on “positive determination” and “courage and creativity” demonstrated lower initial engagement but a steadier use of the platform over time. The findings of this study demonstrate an opportunity for digital mental health interventions to be tailored to individual characteristics in a way that would promote greater initial and ongoing engagement.

## Introduction

Youth mental health is a major global concern, with 75% of psychiatric diagnoses emerging by the age of 25 years [1-3]. Mental illness in adolescence can produce severe disruptions in the social and vocational transition into adulthood [4], with life-long consequences, including impaired social functioning, loneliness, poor educational attainment, and unemployment. The effects of early onset mental illness are of huge consequence to young people and their families, as well as to the economy, estimated to cost approximately US $387 billion per year [5].

Despite a significant proportion of young people living with mental health problems, access to treatment remains limited [6]. In Australia, demand for youth mental health services has resulted in wait times of over 100 days [7], with many young people never attaining access to evidence-based interventions [4]. Prolonged wait times are associated with symptom deterioration, maladaptive and risky coping behaviors, and diminished help-seeking [7,8]. Ubiquitous mobile and internet access in recent years means that digital interventions provide an excellent opportunity to mitigate the global mental health burden by enabling timely, scalable, and low-cost treatment [9-11]. Early evidence shows that digital mental health technologies have similar efficacy to face-to-face treatments [12], significantly improving psychiatric symptom severity [13], social functioning [14], vocational prospects [15], and medication adherence [16], resulting in fewer hospital admissions [15] and demonstrating cost-effectiveness [11].

Digital mental health interventions offer a promising and cost-effective avenue for young people to receive mental health support. Yet, while user engagement is thought to be required for users to experience the full clinical benefits, these interventions often suffer from notable rates of attrition [17]. Although attrition is also a problem that impedes the effectiveness of face-to-face therapy, with a large-scale meta-analysis indicating an average dropout rate of 19.7% across different face-to-face therapies [18], attrition rates are higher in digital interventions. A recent systematic review showed that app-based interventions demonstrate the highest rates of attrition (54.67%), followed by web-based interventions (28%), telehealth (29%), and combination-delivered therapy (26.83%) [19]. These findings indicate that attrition is particularly high when no human support is available. Indeed, Buelens et al [17] found that subjects who enrolled in a purely self-help program showed the fastest decrease in engagement (mean=16.33 days) relative to blended (mean=79.46 days) and guided (mean=80.72 days) treatment paths. Discontinuation may be explained by positive outcomes, such as an improvement in symptoms or continued independent engagement in therapeutic techniques without a requirement for the platform [20]. More commonly, though, attrition is due to unsatisfying user experience, technological barriers, or fluctuations in motivation to conduct effortful tasks [21-24].

With these considerations in mind, moderated online social therapy (MOST) was iteratively co-developed with a team of clinicians, young people, designers, and software engineers in Australia, incorporating a decade of youth feedback and usage data [25-27]. The blended digital intervention integrates clinical care and career support for young people aged 12‐25 years with therapeutic content and a supportive online community of other young people with similar mental health experiences. The platform recommends evidence-based content to users based on their responses to an initial assessment and provides optional tailoring from a clinician—an approach that has previously been shown to increase engagement with digital services [28]. The shared, secure, and private social network was designed to further address attrition rates by enhancing social support and connectedness. Indeed, the use of the social network was identified as a key driver of long-term engagement with the intervention [29]. A recent national-scale evaluation of the MOST platform found that 55% of users were still engaged after 6 weeks and 40% by 12 weeks [30]—a notable improvement on other mental health apps for depression and anxiety that show a lower retention rate of 0.5% to 28.6% after 6 weeks [31]. Nevertheless, ongoing research is required to determine how mental health platforms such as MOST can be tailored to enhance user experience and ensure long-term engagement.

The Persuasive Systems Design (PSD) framework offers a set of guidelines for developers to design and tailor technology in a way that motivates and supports users with the goal of promoting positive changes in behavior and mental state [32]. The PSD framework is built around a comprehensive set of design principles and persuasive strategies categorized into four main components: (1) primary task support, focusing on how technology can help users achieve their goals on the app; (2) dialogue support, encouraging engagement through interactions that motivate and persuade users to persist in achieving their goals; (3) system credibility support, building trust and ensuring the technology is perceived as reliable and competent; and (4) social support, facilitating social influences, through social networks, to encourage sustained behavior change. The framework proposes personalization as a technique to align the technology with users’ needs, interests, and personalities to enhance user engagement and motivation. Recent studies have found customizing different aspects of digital interventions to be advantageous, including symptom monitoring [33], digital reminders [13,34,35], therapeutic content [36,37], delivery approach [38], and interface appearance [33]. As a result, there has been a growing focus on examining how individual characteristics can be used to inform the customization of platforms to enhance their appeal and long-term use [24].

User characteristics, including symptom severity [24,39-41], education level [42,43], gender [44,45], and personality traits [46-48], have been shown to influence engagement with digital mental health platforms. A number of studies have reported that a user’s personality can predict their willingness to engage with an online platform [46-49]. For instance, those who scored higher on extraversion are shown to prefer in-person therapeutic sessions to web-based mental health services. Given that digital platforms inherently lack the interpersonal dynamics of face-to-face interactions, for these users, a social network may enhance the appeal of this type of intervention.

While conceptually similar to personality traits, character strengths are a set of positive thoughts, feelings, and behaviors that are assessed using the Values in Action (VIA) Character Strengths Questionnaire upon enrollment with MOST. Results from this questionnaire are presented to the user, providing them with an assessment of positive psychological attributes to promote self-efficacy, hope for the future, and intrinsic motivation—all of which are associated with sustained interest and improved psychological outcomes [50-52]. Similar to personality traits, character strengths may influence both the duration and nature of engagement with digital mental health interventions. For example, character strengths such as perseverance or intrinsic motivation could influence the duration of engagement, while socially oriented strengths might determine which features of the platform (eg, the social network or peer support) users are more likely to interact with. Assessing users’ character strengths may help tailor digital mental health interventions to individual needs, potentially enhancing engagement and effectiveness. For example, users who score highly on social intelligence may respond better to content focused on social scenarios or interpersonal skills, while those with high levels of creativity or curiosity might be more engaged by interventions that involve novel problem-solving tasks or exploratory learning opportunities.

In this study, we investigated whether a user’s character strengths determined their pattern and duration of engagement with the MOST platform and, in turn, predicted their mental health outcome. Specifically, we aimed to investigate whether scores on each character strength predicted (1) the duration of engagement with MOST, (2) engagement with each component of the MOST platform, and (3) symptom outcome as a function of engagement (the full study is preregistered in OSF Registries [53]). Engagement in this study was quantified as time spent on the platform, number of sessions, number of reactions on the social network, and messages sent to the clinical team in the first 12 weeks following onboarding. Symptom outcome was defined as a user’s score on the Kessler Psychological Distress scale (K10) at weeks 6 and 12, while controlling for baseline.

## Methods

### The MOST Platform

The MOST platform integrates (1) web-based therapeutic content (journeys), (2) web-based social networking, and (3) support from the moderation team (clinicians, peer workers, and career consultants) for young people aged 12‐25 years [25-27,54]. When this paper was written, MOST was available in participating youth mental health services, and young people are referred by their treating clinicians across phases of care—from entry and while on waitlists, concurrent with face-to-face treatment, and following discharge [25,30]. Young people can also be referred to MOST from a service if they are deemed unsuitable for that service (known as “subthreshold”). Young people seeking or receiving mental health support who are referred to MOST complete an initial onboarding assessment that includes questions about their demographics, as well as an adapted 60-item version of the VIA Character Strengths Questionnaire [55]. Users receive an invitation to complete optional self-report questionnaires regarding their mental health at week 0 and again at weeks 6, 12, 18, and 24. Optional self-report mental health questionnaires include the K10 [56].

After enrollment, users could opt in for a brief phone call with a mental health clinician to review their needs and tailor the therapy journey accordingly. MOST’s therapeutic online guided journeys provide support for general anxiety, social anxiety, depression, insomnia, and social functioning. Each journey includes 20 to 40 digital activities that address key drivers of symptoms, functioning, and well-being, such as mindfulness, self-compassion, cognitive restructuring, behavioral activation, and exposure.

Clinicians contact users on a weekly to fortnightly basis via a combination of direct messages, phone calls, and SMS. For users who require vocational support, a career consultant provides individualized assistance, such as identifying options for tertiary study or identifying suitable job openings, supporting specific job-seeking activities, preparing for a job interview, and encouraging the use of their personal strengths. The social network is moderated and led by peer workers—young people who identify as having had lived experience of mental health problems and who are used and trained to provide support on the MOST platform. The social network was designed for young people using the platform to communicate and facilitate social connectedness. Users can post comments or like, react, or respond to comments posted by other young people.

The data analyzed in this study were from young people who enrolled in MOST after August 2021, when data collection started, and before July 2023, when the data were extracted for analysis. Using data from all enrolled participants in this time frame, the sample size was determined to be 6967. Inclusion criteria were (1) help-seeking individuals assessed by a MOST participating youth mental health service, (2) aged 12-25 years, and (3) individuals who provided consent to the platform’s terms of service or, in the case of users aged 12-14 years, have obtained consent from a parent or guardian.

Previous research suggests that user uptake following enrollment cannot be assumed, especially within the context of a digital intervention, and was therefore defined in this study as activity on the platform for at least 1 day [57]. Activity, and therefore user uptake, included completion of the demographic and clinical survey, as well as the character strengths questionnaire. Individuals who did not meet this criterion were excluded from analyses, as no usage data (eg, logins, module access, and clinical interactions) were available. As such, imputing missing data from this group would not be feasible.

### Primary Outcome

The primary outcome variable in this study was user engagement over the first 12 weeks following enrollment. Engagement was operationalized using four continuous metrics: (1) time (minutes) on the platform, (2) sessions on the platform, (3) reactions on the MOST social network, and (4) messages with the clinical team. It is well documented that digital interventions are heavily used following onboarding, and use decreases over time [57]. Hence, both the time spent on the platform and the number of sessions were reported to ensure that we could distinguish users who completed infrequent but longer sessions from individuals who regularly use the platform. The time on platform metric, defined as any time spent on the MOST platform, including viewing therapeutic journeys, direct messaging with the moderation team, and activity on the social network. Time on platform was measured based on sessions. Sessions were defined as at least two consecutive events or actions on the platform (eg, page visits) that were no more than 30 minutes apart, following standard conventions for idle time. The duration of each session was calculated as the elapsed time between the first (session start) and last event (session end) of the session. While this method did not impose a strict maximum time limit for individual sessions, the 30-minute idle time window limits the inclusion of prolonged periods of inactivity. Reactions on the MOST social network were defined as any activity on the social network, including creating a post, posting a comment or liking, responding, or reacting to a comment that someone else had posted. While all participants enrolled with MOST were offered contact with clinicians, responding to messages was optional, and the degree of contact (ie, the number of messages sent) with the clinical team was determined by each individual user. Clinical support has previously been shown to increase the duration of engagement with digital interventions [17,24] and was therefore included as an engagement metric in this study, defined as the number of messages sent to clinicians, peer workers, and vocational workers.

### Independent Variables

#### Overview

The independent variables in this study were (1) character strengths and (2) psychological distress, measured using the K10 [56] at 6 weeks and 12 weeks.

Character strengths were assessed using the 60-item VIA Character Strengths Questionnaire—a validated self-report instrument that measures 24 character strengths across 6 core virtues (justice, temperance, courage, humanity, wisdom, and transcendence). Users rated items on a 5-point Likert scale, generating continuous scores for each individual strength. These scores were analyzed as continuous variables.

Psychological distress was measured using the K10, a 10-item self-report measure assessing symptoms of anxiety and depression. Responses are scored on a Likert scale, with higher scores indicating greater distress. The K10 was treated as a continuous variable in all analyses.

#### Covariates

Age, pronouns, treatment stage, and baseline K10 scores were included as covariates in all models. Age and baseline K10 scores were analyzed as continuous variables, while pronouns and treatment stage (eg, waiting, receiving, unknown, approaching discharge, subthreshold discharge, and discharged) were treated as categorical variables in all analyses.

### Factor Structure of Character Strengths and Statistical Analysis

Original character strength classifications were theory-informed [55]. However, factor analytical studies have converged on a 3- to 5-factor solution to best explain the item-level responses in the VIA Character Strengths Questionnaire [58-63]. Since this study used a short-form adaptation of the questionnaire (consisting of 60 items) with a sample of adolescents experiencing mental health problems, an exploratory factor analysis was used using maximum likelihood estimation to establish the factor structure. A Kaiser-Meyer-Olkin test was used prior to conducting the factor analysis and confirmed factor adequacy (overall Kaiser-Meyer-Olkin test=0.95). Bartlett’s test of sphericity was used to test the null hypothesis that the variables in the correlation matrix are unrelated and unsuitable for a factor analysis. Results from this test were statistically significant (*P*<.001), confirming that the correlation matrix was not an identity matrix.

A factor analysis on the 60-item questionnaire was conducted using an orthogonal rotation (equamax) due to low correlations between the factors (*r*<0.3 [64]). Cattell’s criterion was used to determine the number of factors to extract, whereby a sharp transition or “elbow” in the scree plot where eigenvalues begin to level out indicated that additional factors would add relatively little to the information extracted. The plot was analyzed using the Cattell-Nelson-Gorsuch (CNG) test, an objective implementation of this criterion that computes the slope and determines the point at which there is the greatest transition from horizontal to vertical. The CNG test suggested that a 3-factor structure best explained item-level responses in the dataset.

### Primary Analyses

#### Character Strengths and Time-to-Dropout

An accelerated failure time model was used to investigate whether scores on the 3 character strength factors accelerated or decelerated time-to-dropout, calculated as the number of days from onboarding to the day users were last actively engaged with MOST. Data were right censored for users still engaged in MOST at the time of data extraction. Age, pronouns, treatment stage (ie, waiting for treatment, receiving treatment, approaching discharge, subthreshold discharge, discharged, or treatment stage unknown), and K10 score at enrollment were included in the model, and numeric variables were log-transformed to ensure the comparability of resultant coefficients. Weibull, exponential, Log-Normal, and Log-Logistic distributions were compared, and the Akaike Information Criterion for each was used to identify the best-fitting model. The Log-Normal distribution yielded the lowest Akaike Information Criterion value and was considered the best fit for the data. Model diagnostics were performed by examining residual plots, which indicated that the Log-Normal model fit the data adequately. The accelerated failure time model was exploratory and goes beyond our preregistration [53].

#### Character Strengths and Week-by-Week Platform Engagement

Following this, we used linear mixed-effects models to further examine the relationship between character strengths and platform engagement on a week-by-week basis for the first 12 weeks following onboarding. A separate model was used to assess each of the engagement metrics independently (time spent on the platform, number of sessions, number of reactions on the social network, and messages sent to the clinical team), with week number and scores on each of the 3 character strength factors, as well as age, pronouns, treatment stage, and onboarding K10 score entered as predictors. Measurements of engagement metrics, age, and K10 scores were log-transformed to enable comparability of resultant coefficients. All 3 models included a random intercept and slope to account for individual differences and the rate of change over time.

### Mediation Analysis

Our preregistration stated that a path analysis would be used to investigate whether scores on the character strength factors predict change in mental health symptoms between baseline and 12 weeks as a function of platform engagement. However, user completion of mental health questionnaires (eg, K10) was optional following enrollment, and the amount of missing data at the 12-week follow-up was substantial. Furthermore, missingness was not at random, which prohibited multiple imputation. Specifically, the percentage of missing values across the variables in the dataset varied between 0% and 84%. In total, 6579 out of 6967 records (94%) were incomplete. Missing data were highest for K10 scores—our primary outcome—at baseline (58%), 6 (80%) weeks, and 12 (84%) weeks. A logistic regression model was used to investigate whether K10 scores at baseline and 6 weeks were associated with missing data at week 12 (0 for not missing and 1 for missing). The overall model was statistically significant compared to the null model (*χ*^2^_1411_=1861.5; *P*=.02) and demonstrated that a 10% increase in K10 scores at 6 weeks was associated with a 2% decrease in missing data at week 12 (95% CI 0.96-0.99; *P=.*006) and that a 10% increase in baseline K10 score was associated with a 2% increase in missing data at week 12 (95% CI 1.01-1.04; *P=.*01). These results indicate the missing data were influenced by the severity of mental health symptoms, that is, not missing completely at random. While a missing at random mechanism allows for imputation, the extremely high rate of missingness at weeks 6 and 8 may limit the reliability of imputed estimates. We, therefore, determined that the preregistered path analysis would not be appropriate. We discuss the implications of missing data in more detail in the Discussion section.

All analyses were performed in RStudio (Posit, PBC) using the following packages: *psych* (version 2.3.12), *lme4* (version 1.1‐34), and *survival* (version 3.5‐7). Code for the full analysis is available on GitHub [65].

### Deviations From Preregistration

As outlined in the Statistical Analysis section, there were 2 deviations from our preregistered analysis plan [53]. First, we did not conduct the planned path analysis due to substantial missing data for the outcome variable, which would have compromised the validity of the results. Second, we added an exploratory accelerated failure time model to further investigate the relationship between scores on the character strengths and time-to-dropout. This addition was not prespecified but was deemed appropriate given the nature of the data available.

### Ethical Considerations

Ethical approval for the use of data in this study was granted by the Royal Melbourne Hospital Human Research Ethics Committee (HREC/83853/MH-2022). All data analyzed were obtained from participants who provided informed consent at the time of enrollment, permitting the use of their information for research purposes. While MOST collects personal information, all data are deidentified upon extraction, prior to analysis, to ensure participant confidentiality. As part of a quality improvement initiative, MOST compensates users for their participation. Participants received Aus $20 (US $13.10) for completing questionnaires at baseline, 6 weeks, and 12 weeks.

MOST incorporates a comprehensive safety protocol to ensure a secure and supportive environment for users, particularly young people. The platform is hosted on Amazon Web Services, with data security and identity management by Orygen Digital’s engineering department in accordance with national standards that meet Australian research and data protection requirements. The platform and database are protected against unauthorized access through a range of technical safeguards aligned with industry best practices, including those outlined by the Open Web Application Security Project. Privacy and online safety protocols are informed by guidelines from the Australian Communications and Media Authority.

During onboarding, a MOST clinician provides users with an orientation that outlines the platform’s terms of use and guidelines for safe engagement. Users must agree to these terms prior to accessing the system and are supported with guidance on appropriate usage when needed. Users are also asked to nominate an emergency contact.

MOST’s social networking features are actively moderated. The platform includes a reporting function that enables users to flag concerning content. Reports are reviewed by trained moderators who may remove content or restrict or deactivate accounts if necessary. Users have the option to hide their profiles and activities to enhance personal privacy and safety.

## Results

### Demographics and Descriptive Statistics

The data presented in this paper is from the 6967 young people who enrolled with MOST between August 2021 and July 2023 and provided consent for their data to be used for research purposes. The demographic characteristics of this sample are presented in Table 1.

Mean K10 score was 34.47 (SD 8.28; N=2958) at enrollment, 32.19 (SD 8.24; n=1879) at week 6, and 31.70 (SD 9.33, n=1454) at week 12 (Figure S1 in Multimedia Appendix 1). A repeated measures ANOVA was performed to examine K10 scores over the 12-week period. Results revealed a significant effect of time on K10 score (*F*_2,1745_=56.58; *P*<.001). Tukey post hoc comparisons further explored pairwise differences in K10 scores between time points. K10 scores significantly decreased between onboarding and week 6 (*P*<.001), onboarding and week 12 (*P*<.001), and weeks 6 and 12 (*P*<.001). Mean scores on all additional mental health questionnaires are presented in Table S1 in Multimedia Appendix 1.

**Figure 1.**
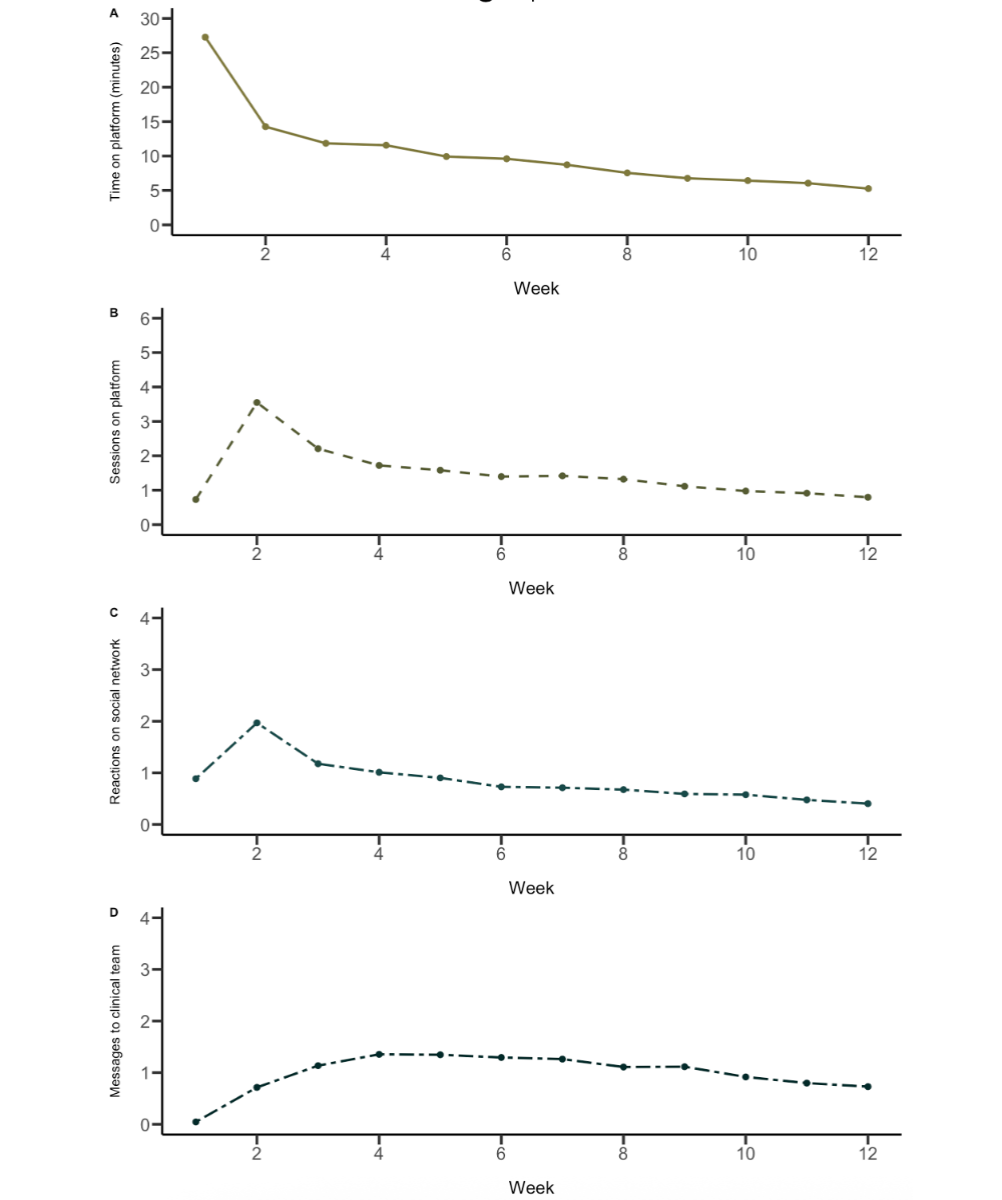
MOST 12-week engagement patterns. (**A**) Mean time on platform, (**B**) sessions on platform, (**C**) reactions of the social network, and (**D**) messages sent to the clinical team, measured week-by-week for the first 12 weeks from enrollment. MOST: moderated online social therapy.

On average, users remained engaged with the platform for 72.64 (SD 106.64) days, with 14.22 (SD 32.09) of those days spent actively using the platform (with activity defined as any page view, session, or action on the platform). Figure 1 illustrates user engagement with the MOST platform in terms of the amount of time users spent on the platform, number of sessions on the platform, reactions on the social network, and interactions with the clinical team, measured on a week-by-week basis for the first 12 weeks following enrollment. Users spent an average of 126.23 (SD 500.04) minutes on the platform, logged in for 17.72 (SD 39.34) sessions (defined as a period of activity that included more than 1 page visit with no more than 30 minutes of inactivity between visits), reacted 11.54 (SD 39.58) times on the social network (ie, posted a comment or liked, responded, or reacted to a comment that someone else had posted on the social network), and contacted the clinical team 11.79 (SD 27.66) times in the first 12 weeks.

**Table 1.** Sample demographics (N=6967).

	Values
Age (years), mean (SD)	17 (3.3)
Pronouns, n (%)	
She or Her	4591 (66)
He or Him	1445 (21)
They or Them	746 (11)
Else	185 (3)
Indigenous status, n (%)	
Aboriginal	144 (2)
Torres Strait Islander	8 (<1)
Aboriginal and Torres Strait Islander	7 (<1)
Nonindigenous	2248 (32)
Unknown	4560 (65)
Treatment stage, n (%)	
Waiting	3439 (49)
Receiving	1504 (22)
Unknown	1090 (16)
Approaching discharge	626 (9)
Subthreshold discharge	299 (4)
Discharged	9 (<1)

### Factor Structure of Character Strengths

**Figure 2.**
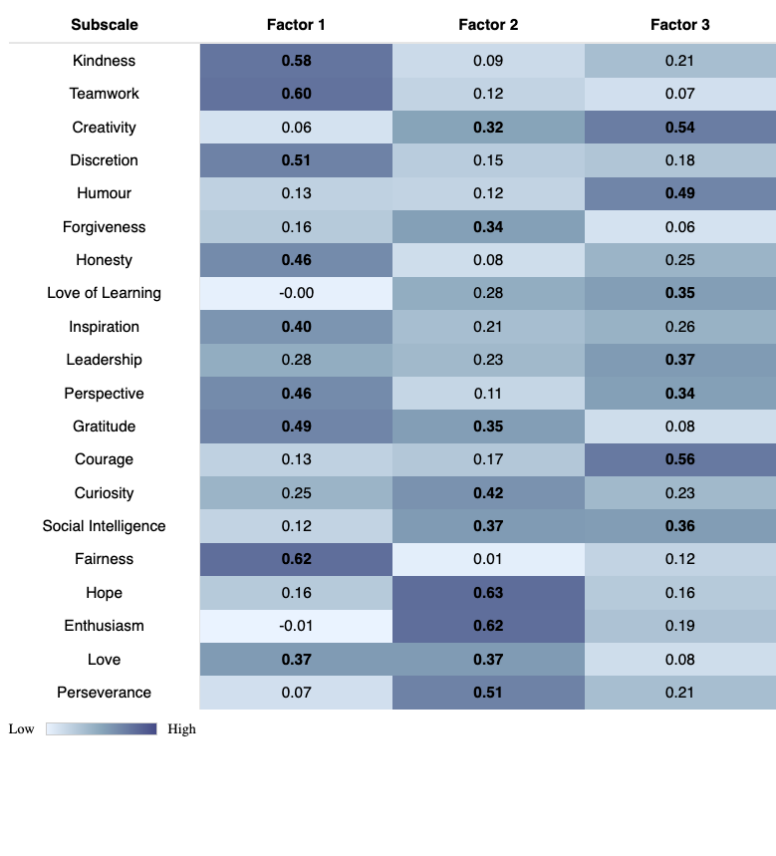
Factor loadings. Loadings from a factor analysis on the VIA Character Strengths Questionnaire. Each column corresponds to a factor. Loadings indicate the strength and direction of the relationship between the character strength subscale and the respective factor. Higher loadings suggest a stronger association. Cell shading reflects loading magnitude, with darker blue indicating a higher loading. Loadings greater than 0.3 are in bold to highlight the dominant subscales, aiding in the interpretation of the underlying latent factors. Based on these loadings, factors were labeled (1) “social harmony,” (2) “positive determination,” and (3) “courage and creativity.” Specifically, for social harmony (factor 1), the teamwork, kindness, and discretion subscales were most heavily weighted; positive determination (factor 2) was heavily weighted by the hope, enthusiasm, and perseverance subscales; and courage and creativity (factor 3) was weighted predominantly by the courage, creativity, and humor subscales. VIA: Values in Action.

Responses to the 60 VIA Character Strength Questionnaire items were entered into a factor analysis. Both a scree plot and CNG test indicated that a 3-factor solution best explained the variance in the item-level responses in this dataset (Figure S2 in Multimedia Appendix 1). Factors were labeled “social harmony,” “positive determination,” and “courage and creativity” based on the strongest individual item loadings (Figure 2). Specifically, “social harmony” was defined predominantly by traits of fairness, teamwork, kindness, and discretion; “positive determination” was defined by traits of hope, enthusiasm, and perseverance; and “courage and creativity” was defined by courage, creativity, and humor. These factors were comparable with the other 3-factor models produced in the personality literature, which typically report a social factor, a personal agency factor, and a conscientiousness factor, with similar component loadings, respectively [66].

### Primary Analysis

#### Character Strengths and Time-to-Dropout

An accelerated failure time model was used to investigate whether higher scores on each of the character strength factors accelerate or decelerate time-to-dropout, defined as the number of days from enrollment to the day users last actively engaged with MOST. Scores on the social harmony, positive determination, and courage and creativity factors were entered into a single model, as well as age, treatment stage, pronouns, and baseline K10 score. Age and K10 scores were log-transformed to aid interpretation of the results.

A higher score on the social harmony factor was associated with an accelerated dropout rate (coefficient=−0.15, 95% CI −0.26 to −0.04; *P*=.008), indicating that individuals who scored higher on social harmony character strengths disengaged with the digital platform more rapidly. Scores on the positive determination factor and courage and creativity factor did not show significant effects on dropout rate (positive determination factor: coefficient=0.09, 95% CI −0.01 to 0.19; *P*=.09 and courage and creativity factor: coefficient=−0.02, 95% CI −0.11 to 0.07; *P*=.66).

Treatment stage was also predictive of time to drop out, with individuals who were receiving face-to-face treatment, and whose treatment stage was unknown demonstrating an accelerated dropout rate (face-to-face treatment: coefficient=−0.60, 95% CI −1.01 to −0.19; *P*=.004 and unknown treatment stage: coefficient=−1.46, 95% CI −1.87 to −1.05; *P*<.001). Age, pronouns, and K10 score at enrollment were not related to time-to-drop out.

#### Character Strengths and Week-by-Week Platform Engagement

**Figure 3.**
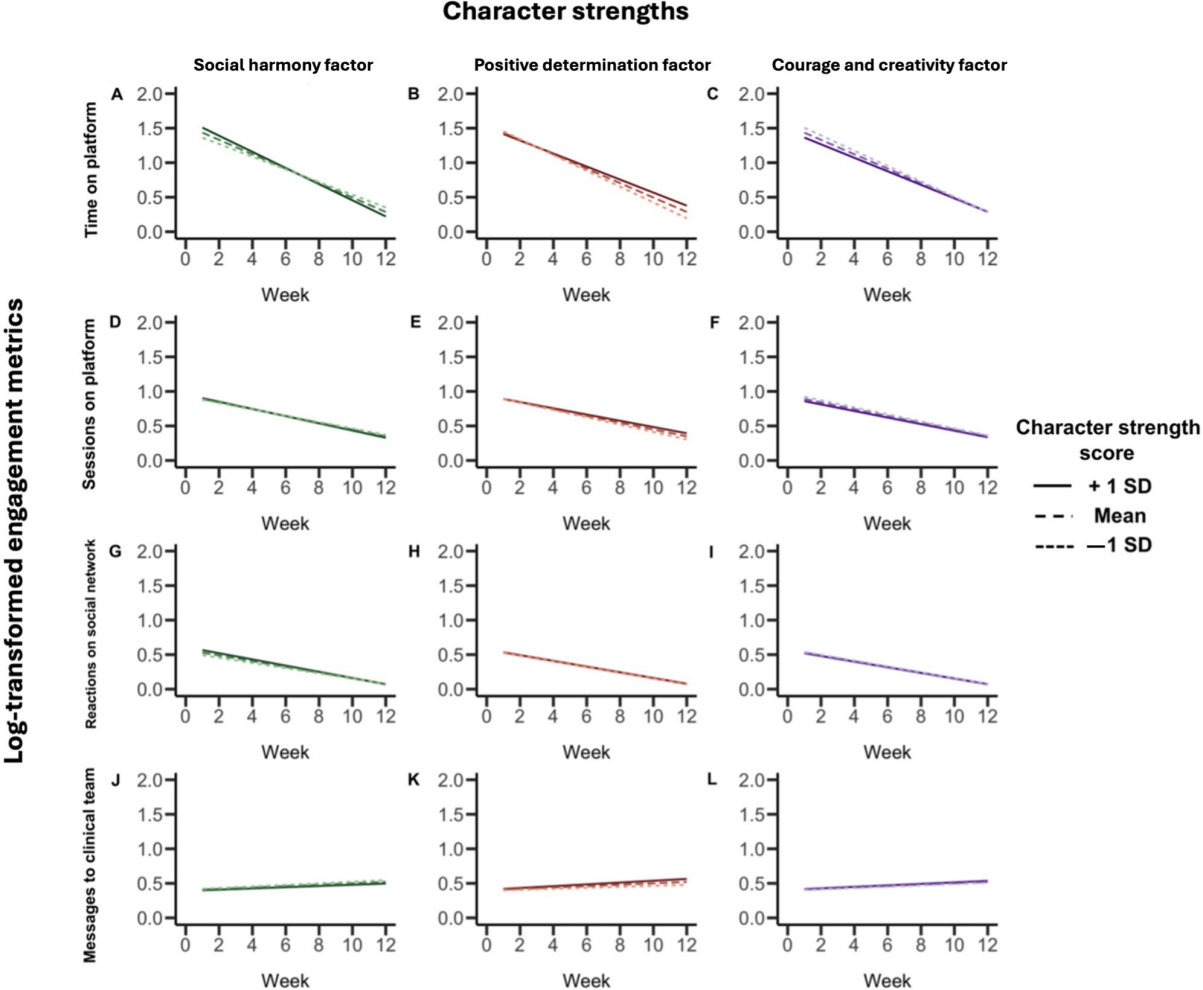
Character strengths and week-by-week engagement. Interaction effects derived from linear mixed effects models investigating the relationship between character strength factors and week-by-week platform engagement for the first 12 weeks since onboarding. The x-axis represents the week number, and the y-axis represents the log-transformed engagement metric; (**A-C**) time on platform, (**D-F**) sessions on platform, (**G-I**) reactions on the social network, (**J-L**) messages to the clinical team. Measurements of engagement metrics were log-transformed to allow comparability of the resultant coefficients. The solid lines represent the mean score on the character strengths factor, while the dashed lines represent 1 SD from the mean. The interaction effect is visualized by the divergence or convergence of the mean and the dashed lines over time.

Linear mixed-effects models were next used to investigate whether character strength factors were associated with changes in each engagement metric (time on platform, number of sessions, reactions on the social network, and interactions with the clinical team) week-by-week over 12 weeks. To aid the interpretation of the time-by-character strength interaction, mean-centered continuous character strength variables were plotted (Figure 3). Plots of mean±1 SD depict the model-implied relationship for each character strength.

### Time Spent on Platform

There was a significant interaction effect between scores on social harmony and week number, indicating that the effect of character strength on time spent on the platform (defined as any time spent on the MOST platform, including viewing therapeutic journeys, direct messaging with the moderation team, and activity on the social network) changed over the 12-week period (Figure 3A; β=−.01; SE 0.00; *t*_2748_=–5.05; *P*<.001). Specifically, for individuals scoring higher on this factor, while time spent on the platform was initially greater, the rate of decline in platform usage over the 12 weeks was more pronounced. The opposite was true for the positive determination factor, whereby individuals scoring higher on this character strengths factor initially spent less time on the platform but demonstrated a slower decline in platform use over the 12 weeks (Figure 3B; β=.01; SE 0.00; *t*_2748_=4.05; *P*<.001). For courage and creativity character strengths, a significant interaction effect with week number suggested that individuals scoring higher on this character strength factor spent less time on the platform, and the rate of decline in time spent on the platform over the 12 weeks was less pronounced (Figure 3C; β=.01; SE 0.00; *t*_2748_=2.66; *P*=.008). Age (*t*_2737_=1.39; *P*=.16), pronouns (all *P*>.05), and K10 score at enrollment (*t*_2737_=–1.47; *P*=.14) were not related to time spent on the platform. However, individuals whose treatment stage was unknown spent a greater amount of time on the platform over the 12-week period (β=.36; SE 0.08; *t*_2737_=4.68; *P*<.001).

### Number of Sessions on Platform

A significant interaction effect between scores on social harmony and week number indicated that while higher scores on this factor were associated with greater number of weekly sessions (defined as a period of activity that included more than 1 page visit with no more than 30 minutes of inactivity between visits) soon after enrollment, the rate of decline in number of sessions spent on the platform was more pronounced for these individuals (Figure 3D; β=−.00; SE 0.00; *t*_2837_=−2.26; *P*=.02). In contrast, a significant interaction effect between scores on the positive determination factor and week number illustrated that while individuals scoring higher on this factor completed a fewer number of weekly sessions initially, these individuals also demonstrated a slower rate of decline in the number of sessions over the 12 weeks (Figure 3E; β=.00; SE 0.00; *t*_2837_=3.02, *P*=.003). While scores on courage and creativity were unrelated to the rate of change in the number of sessions on the platform over the 12 weeks (Figure 3F; β=−.00; SE 0.00; *t*_2837_=−1.26; *P*=.21), a significant main effect of courage and creativity scores on the number of sessions spent on the platform indicated that higher scores on this character strength were associated with fewer overall sessions on the platform (β=−.03; SE 0.01; *t*_2863_=−2.64; *P*=.008).

Age (*t*_2826_=−0.06; *P*=.95), pronouns (all *P*>.05), and K10 score at enrollment (*t*_2826_=−1.02; *P*=.31) were not related to the number of sessions on the platform. Individuals whose treatment stage was unknown were more engaged with the platform, demonstrated by a greater number of sessions over the 12-week period (β=.13; SE 0.05; *t*_2826_=2.87; *P*=.004).

### Use of the Social Network

We did not find evidence for interaction effects between week number and scores on social harmony (Figure 3G; β=−.00; SE 0.00; *t*_1092_=−1.80; *P*=.07), positive determination (Figure 3H; β=−.00; SE 0.00; *t*_1092_=−0.01; *P*=.99), or courage and creativity (Figure 3I; β=.00; SE 0.00; *t*_1092_=0.26; *P*=.80), suggesting that scores on these factors were not predictive of the rate of decline in use of the social network (with activity on the social network defined as posting a comment or liking, responding, or reacting to a comment that someone else had posted on the social network). In addition, we did not find evidence for a main effect of scores on social harmony (β=.04; SE 0.02; *t*_1252_=1.89; *P*=.06), positive determination (β=.00; SE 0.02; *t*_1179_=0.23; *P*=.82), or courage and creativity (β=−.01; SE 0.02; *t*_1113_=−0.47; *P*=.64) on the number of reactions posted on the social network. Nevertheless, a significant main effect of week number indicated an overall decrease in the use of the social network reactions over 12 weeks across individuals (β=−.04; SE 0.00; *t*_1092_=−20.84; *P*<.001). Treatment stage (all *P*>.05), age (*t*_1082_=−1.99; *P*=.05), pronouns (all *P*>.05), and K10 score at enrollment (*t*_1082_=−1.48; *P*=.14) were not related to the use of the social network over the 12-week period.

### Messages With the Clinical Team

We did not find evidence for interaction effects between week number and scores on social harmony (Figure 3J; β=−.00; SE 0.00; *t*_1429_=−0.79; *P*=.43), positive determination (Figure 3K; β=.00; SE 0.00; *t*_1429_=1.94; *P*=.053), or courage and creativity (Figure 3L; β=.00; SE 0.00; *t*_1429_=0.43; *P*=.67), suggesting that scores on these factors were not predictive of a change in the number of messages sent to the clinicians. In addition, we did not find evidence for a main effect of scores on social harmony (β=−.01; SE 0.01; *t*_1596_=−0.65; *P*=.52), positive determination (β=.01; SE 0.01; *t*_1518_=0.54; *P*=.59), or courage and creativity (β=.01; SE 0.01; *t*_1440_=0.50; *P*=.61) on the overall number of messages sent to the clinical team. In contrast to the other platform engagement metrics, a significant main effect of week number indicated an overall increase in the number of messages sent over the 12 weeks (β=.01; SE 0.00; *t*_1429_=6.55; *P*<.001). Furthermore, a significant main effect of age (β=.28; SE 0.07; *t*_1419_=3.82; *P*<.001) suggested that older individuals had a greater number of interactions with the clinical team over the 12 weeks. Treatment stage (all *P*>.05), pronouns (all *P*>.05), and K10 score at enrollment (*t*_1419_=1.87; *P*=.06) were not related to the number of interactions with the clinical team.

## Discussion

### Principal Findings

In recent years, digital mental health interventions have generated significant public interest as an accessible and cost-effective treatment avenue for mental health problems [11,67-69]. However, engagement remains an area of concern for these interventions. Indeed, a recent study found that only 3% of users still opened mental health apps 1 month after download [70]. Adjusting digital platforms according to user needs is a key approach to ensuring user engagement and effectiveness. In this study, we examined whether users’ scores on 3 character strength dimensions—“social harmony,” “positive determination,” and “courage and creativity”—were related to patterns of engagement on the MOST digital mental health platform. Our findings revealed that distinct character strengths were associated with dissociable patterns of user engagement.

An accelerated failure time model revealed that both individuals who scored higher on social harmony, a dimension encompassing traits such as fairness, teamwork, kindness, and discretion, and individuals receiving face-to-face therapy were more likely to drop out of MOST at a faster rate. A further analysis showed that those scoring higher on social harmony demonstrated greater initial engagement that declined rapidly over time. In contrast, users with higher scores on positive determination (defined by hope, enthusiasm, and perseverance) and courage and creativity (defined by courage, creativity, and humor) exhibited more moderate initial engagement but maintained more consistent use throughout the first 12 weeks compared to those with lower scores on these character strengths. These findings offer implications for both theory and the design of digital mental health platforms by suggesting that accounting for individual character strengths may support more sustained user engagement.

### Integration With Broader Psychological Theory and Practice

The PSD framework proposes the personalization of digital platforms for individual preferences and needs, to promote user engagement and behavior change [32]. Our findings align with this theory, demonstrating that an individual’s character strengths uniquely influence engagement patterns. Whereas previous studies have provided evidence that personalizing platforms based on one’s personal capacity, level of motivation, and illness severity can enhance engagement [71-73], our results suggest that character strengths should also be considered during personalization. Specifically, there is the potential that matching program content and features to the values (eg, social connection and perseverance) which define each character strength could help to create an experience that feels more personally aligned and motivating for each individual.

The MOST platform is grounded in the self-determination theory of behavior change, which proposes that sustained engagement and intrinsic motivation are driven by the extent to which individuals experience support for 3 basic psychological needs: autonomy, competence, and relatedness [74]. Making sure that the platform content aligns with the individual’s character strengths could help to boost feelings of autonomy and competence and enhance the relatedness of the intervention, thereby supporting behavior change. The MOST platform integrates distinct features such as clinical content, clinician support, peer support, the social network, and career support, which are designed to work cohesively but also allow flexibility to create a personalized experience for each young person. Users scoring high on the social harmony factor showed strong initial engagement but rapid drop-off. For these individuals, who value teamwork and kindness, emphasizing platform features like peer support and the social network or adding peer-led check-ins in the later weeks of engagement could be particularly impactful for keeping individuals engaged beyond the initial weeks. Indeed, the guiding self-determination theory of behavior change would suggest that for those scoring high on Social Harmony, the use of social features could directly contribute to the experience of relatedness by facilitating meaningful connections and shared understanding with others, thus aligning longer-term intervention completion with their personal values. Moreover, as peers share personal experiences and provide mutual aid, regularly reminding users to engage with these aspects of the platform may also promote a sense of personal responsibility and self-determination, leading to more sustained engagement [20,71].

Additionally, our findings suggested that individuals scoring higher on the courage and creativity character strengths engage with the intervention more consistently over time, but their overall level of engagement was modest. To boost overall engagement rates among these individuals, who exhibit strengths such as creativity and humor, incorporating more fun and exploratory elements into the intervention may enhance intrinsic motivation to complete the program. It is well understood that engagement with digital interventions is strongly influenced by user experience, including the ease of use, layout, and visual appearance of a platform [75-78]. Gamifying features, for example, by rewarding progress, setting challenges and goals, using avatars, and incorporating a narrative in the therapeutic content, may be particularly engaging for those high in courage and creativity [79,80]. Providing opportunities for users to offer feedback on the content and platform design over the course of intervention use could also promote engagement. Given their overall modest level of engagement, involving users high in courage and creativity in co-design processes may be particularly effective and ultimately lead to a product that is more aligned with the needs of an underengaged cohort. For this group, co-design offers not just an opportunity to shape content but also to exercise agency and contribute creatively—factors that are closely aligned with their intrinsic values and will promote true co-production.

For individuals who are more intrinsically motivated, such as those scoring higher on positive determination who demonstrate sustained but modest engagement, a measurement-based care (MBC) [81] approach could help to increase platform use by supporting core psychological needs. MBC involves the regular monitoring of an individual’s progress, providing feedback on both positive and negative changes over the course of treatment [81-83]. By making progress visible, ongoing feedback can reinforce a sense of personal achievement and competence, as well as highlighting areas for continued growth. For individuals scoring higher on positive determination, who demonstrate traits of enthusiasm and perseverance, tracking progress could help align their actions with their intrinsic values, enhancing engagement. Furthermore, giving users the ability to adapt and personalize treatment content and strategies in response to these assessments could lead to a greater sense of autonomy over the therapeutic process. Evidence consistently shows that MBC contributes to improved outcomes by ensuring that interventions remain attuned to the evolving needs and priorities of the individual, thereby supporting long-term engagement and improved outcomes [84-86].

Finally, our finding that individuals receiving face-to-face care drop out of MOST at an accelerated rate supports results from previous studies showing that engagement with digital mental health tools is related to the level of clinical contact that a user is receiving [17,19]. It is possible that therapist involvement accelerated recovery, reducing the perceived need to use the digital platform. Although therapist guidance is resource-intensive and may not be feasible for many low-cost digital mental health interventions, several recent studies have demonstrated that peer support can enhance feelings of social connectedness in a manner that mitigates stigma and shame [87,88]. More recently, artificial intelligence (AI) chatbots have been integrated into platforms as scalable, human-like support tools. These chatbots have shown promising results in increasing engagement and improving outcomes; for example, in one study evaluating a smoking cessation app, chatbot use doubled user engagement [89]. These results show that both peer support and AI-based solutions may serve as a viable alternative to clinical contact in sustaining user engagement.

While tailoring platform features to individual user characteristics, such as character strengths, aligns with behavior change theories, our suggestions remain speculative. Future studies are needed to empirically test this assumption and determine whether such personalization results in more sustained engagement with digital health interventions.

### Strengths and Limitations

The findings reported here should be considered in light of the limitations of this study. First, while the large sample size was a considerable strength of this study, offering valuable insights into the relationship between character strengths and engagement with digital mental health interventions, the substantial amount of missing data at the 12-week follow-up precluded our planned analysis of the relationship between engagement patterns and mental health outcomes. Missing data at follow-up time points is a common issue in digital health research and one that prohibits the robust investigation into the effectiveness of digital interventions [90]. Without this data, it is not possible to investigate whether patterns of platform engagement directly translate into meaningful mental health improvements or sustained behavioral change. Previous studies that have investigated the degree of platform engagement that is required for significant decreases in clinical measures have yielded mixed results. The mixed findings might reflect the active versus passive engagement styles of different users [91-94]. A study by Hoffman et al [95] identified 3 clusters of engagement types, differing in terms of behavior, cognitive and affective engagement, as well as the timing of engagement. “Efficient engagers,” who were highest on affective and cognitive engagement, demonstrated the greatest decline in depressive symptoms and stress. This data highlights the importance of measuring active user attendance (eg, clicks on a page and entries into text boxes) rather than passive use of digital platforms.

This leads us to a second limitation of the study concerning the interpretation of engagement metrics. Not all usage data equally reflects meaningful therapeutic engagement. For example, activities such as sending messages to the clinical team or contributing to the social network likely indicate intentional and active use. In contrast, metrics such as total time spent on the platform or number of logins may reflect passive browsing rather than substantive interaction with therapeutic content. This distinction is important, as a recent study demonstrated that use of the MOST social network, in addition to engagement with therapy components, was necessary for users to experience symptom improvements [17]. To better capture active engagement, future studies should incorporate more granular behavioral metrics, such as the number of module completions, text box entries, or clicks within interactive content. However, behavioral metrics alone may not capture the full picture. Engagement is a multidimensional construct that includes experiential and relational factors. Therefore, future research should also assess user-reported measures such as satisfaction, perceived helpfulness, therapeutic alliance, and broader functional outcomes like quality of life and goal attainment. These data would allow for a more comprehensive evaluation of how digital engagement translates into meaningful clinical change.

A third limitation of this study is the exclusion of participants who did not engage with the intervention for at least 1 day. This decision was guided by our primary aim, to examine predictors of engagement with the digital platform, which required a minimum threshold of interaction to ensure that behavioral data analyzed reflected actual use. Consistent with prior research in digital mental health [57], we defined engagement as at least 1 day of activity on the platform, which included completing the demographic and mental health questionnaires, as well as the character strengths questionnaire. Participants who did not meet this criterion were excluded because no usage data (eg, logins, module access, and clinical interactions) were available, and thus imputing missing data for this group was not feasible. While this approach allowed for more robust analysis of engagement predictors, doing so excluded individuals who registered but never initiated use, an important subgroup that likely constitutes a substantial proportion of digital mental health intervention users. Understanding this population may be better suited to implementation science methods or uptake-focused studies that can capture preengagement variables (eg, motivation and referral context), which were beyond the scope of this study.

### Conclusions and Future Directions

The findings of this study suggest that different character strengths predict distinct patterns of engagement over time, with some individuals showing greater initial engagement but an accelerated dropout rate, while others demonstrate more modest initial engagement but a steadier decline in engagement over time. Our results demonstrate the importance of using multicomponent digital mental health interventions with flexible features that can meet the needs of a diverse range of individuals. The substantial decline in user engagement over 12 weeks also highlights the challenges of long-term engagement in digital health interventions and points to the need for more innovative approaches, such as personalization, to enhance sustained engagement.

## Supplementary material

10.2196/73793 Multimedia Appendix 1 Supplementary material.

## Abbreviations

AI: artificial intelligence
CNG: Cattell-Nelson-Gorsuch
K10: Kessler Psychological Distress scale
MBC: measurement-based care
MOST: moderated online social therapy
PSD: Persuasive Systems Design
VIA: Values in Action
