# Supplementary material for: User Character Strengths and Engagement Prediction on a Digital Mental Health Platform for Young People: Longitudinal Observational Study
Source: J Med Internet Res. 2025 Aug 25;27:e73793. doi: 10.2196/73793 (PMC12387396; doi:10.2196/73793)

**Supplementary Materials**

**Figure S1.** Mean K10 scores at onboarding, week 6 and week 12. K10 scores significantly decreased between onboarding and week 6 (*p* < .0001), onboarding and week 12 (*p* < .0001), and week 6 and week 12 (*p* = .0001). Error bars represent the standard deviation.


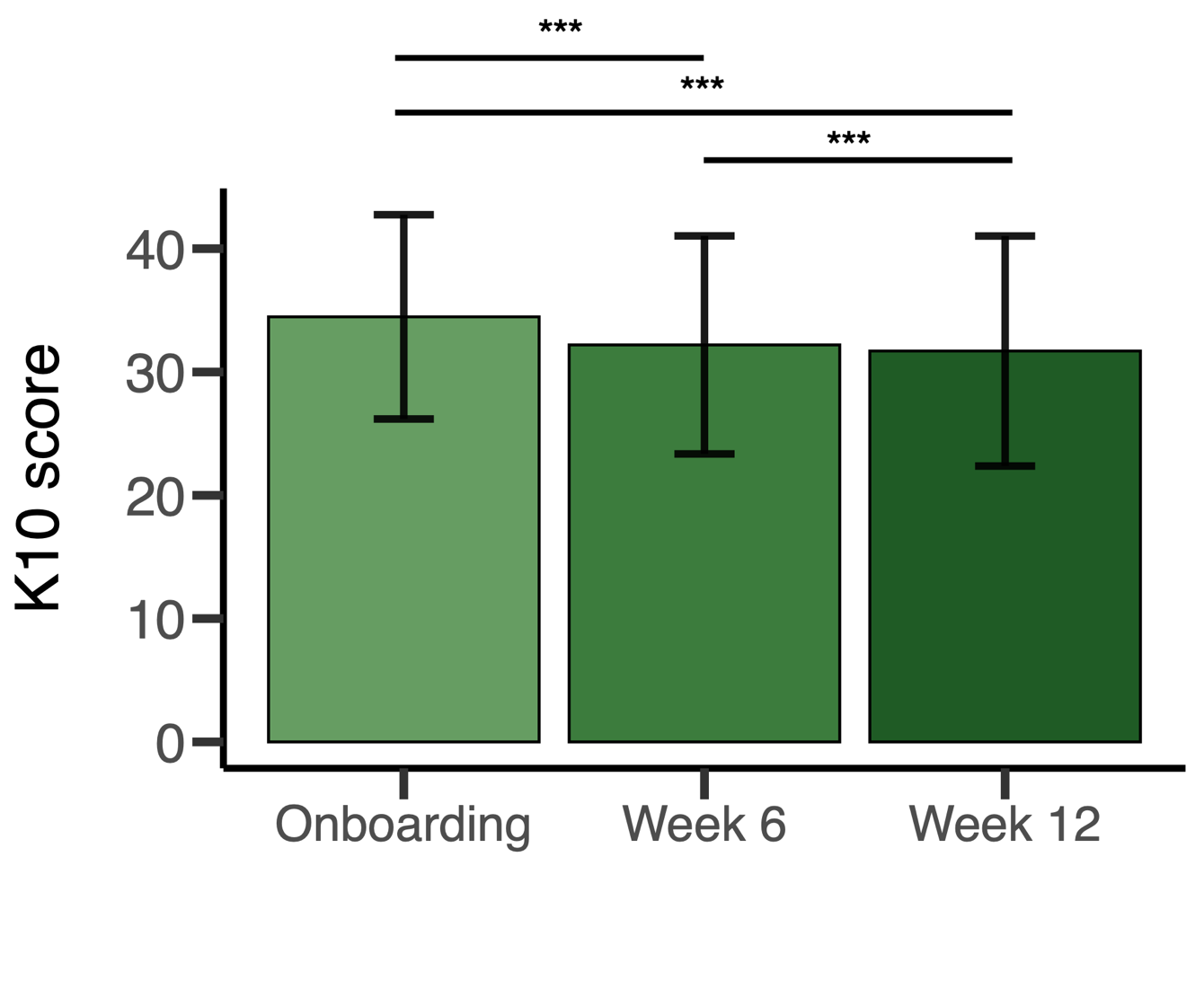


**Table S1.** Mean score and number of participants completing mental health questionnaire measures at onboarding. Patient Health Questionnaire-2, PHQ-2 (36); Generalized Anxiety Disorder-2, GAD-2 (37); Mini Social Phobia Inventory, Mini-SPIN(38); Perceived Stress Scale-4, PSS-4 (39); short Warwick-Edinburgh Mental Wellbeing Scale, SWEMWBS (40); UCLA Loneliness Scale, UCLA-LS (41); Friendship Scale (42); Child Health Utility Instrument, CHU9D (43). Standard deviation, SD.

| Questionnaire (/total score) | Mean (SD) | Frequency |
| --- | --- | --- |
| K10 (/50) | 34.47 (8.28) | 2958 |
| PHQ-2 (/6) | 3.80 (1.68) | 3004 |
| GAD-2 (/6) | 3.94 (1.65) | 3018 |
| Mini-SPIN (/12) | 8.10 (3.25) | 2949 |
| PSS-4 (/16) | 10.09 (2.58) | 2358 |
| SWEMWBS (/35) | 17.87 (4.53) | 2948 |
| UCLA LS (/16) | 6.81 (1.69) | 3010 |
| Friendship Scale (/25) | 11.18 (4.86) | 2390 |
| CHU9D () | 26.49 (7.52) | 1924 |

**Figure S2.** Scree plot of eigenvalues from a factor analysis of the VIA Character Strengths Questionnaire. The plot shows the eigenvalues for all possible factor solutions (N=60). An elbow was identified at factor 3, where Eigenvalues begin to level out.


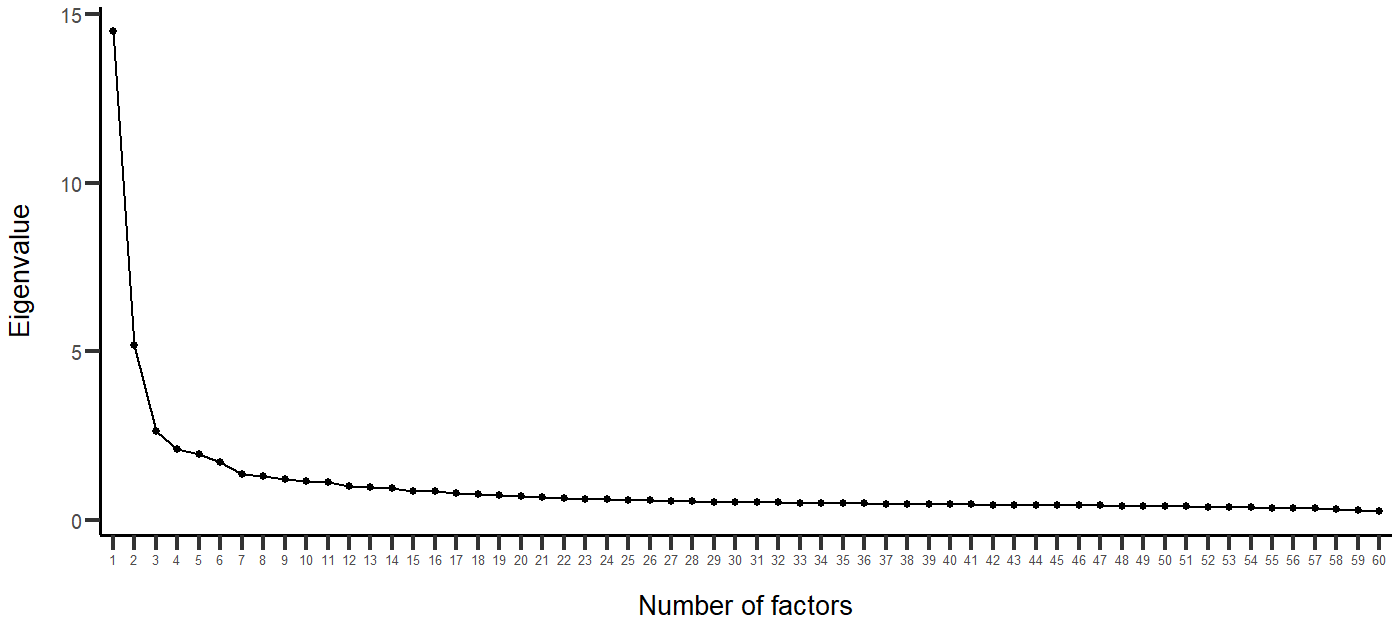

Supplement: Multimedia Appendix 1 [file jmir-v27-e73793-s001.docx]
